# Pharmacological evidence for the folk use of Nefang: antipyretic, anti-inflammatory and antinociceptive activities of its constituent plants

**DOI:** 10.1186/s12906-015-0703-7

**Published:** 2015-06-09

**Authors:** Protus Arrey Tarkang, Faith A. Okalebo, Juma D. Siminyu, William N. Ngugi, Amos M. Mwaura, Jackson Mugweru, Gabriel A. Agbor, Anastasia N. Guantai

**Affiliations:** Centre for Research on Medicinal Plants and Traditional Medicine, Institute of Medical Research and Medicinal Plants Studies (IMPM), P. O. Box 6163, Yaoundé, Cameroon; Department of Pharmacology and Pharmacognosy, School of Pharmacy, University of Nairobi, P. O. Box 19676-00202, Nairobi, Kenya; Department of Veterinary Anatomy and Physiology, Faculty of Veterinary Medicine, University of Nairobi, P. O. Box 30197-00100, Nairobi, Kenya

**Keywords:** Medicinal plants, Nefang, Pharmacological effects, Antipyretic, Anti-inflammatory, Antinociceptive activities

## Abstract

**Background:**

*Nefang* is a polyherbal anti-malarial composed of *Mangifera indica (****MiB*** and ***MiL***; bark and leaf), *Psidium guajava (****Pg****), Carica papaya (****Cp****), Cymbopogon citratus (****Cc****), Citrus sinensis (****Cs****)* and *Ocimum gratissimum (****Og****)* (leaves). Previous studies have demonstrated its in vitro and in vivo antiplasmodial activities, antioxidant properties and safety profile. This study aimed at evaluating the antipyretic, anti-inflammatory and antinociceptive activities of the constituent plants of *Nefang* which are relevant to the symptomatic treatment of malaria fever.

**Methods:**

Antipyretic activities were determined by the D-Amphetamine induced pyrexia and Brewer’s Yeast induced hyperpyrexia methods. Anti-inflammatory activities were investigated using the carrageenan-induced rat paw edema method. Antinociceptive activities were determined by mechanical nociception in the tail pressure and thermal nociception in the radiant heat tail flick and hot plate methods. Data was analysed using the one way ANOVA followed by Neuman-Keuls multiple comparison test.

**Results:**

Best percentage inhibition of induced pyrexia (amphetamine/brewer’s yeast; *p* < 0.05) was exhibited by *Cc* (95/97) followed by *Og* (85/94), *MiL* (90/89), *MiB* (88/84) and *Cs* (82/89). *Cc* and *Og* exhibited comparable activities to paracetamol (100/95).

Anti-inflammatory studies revealed paw edema inhibition (%) as follows (*p* < 0.05): Indomethacin (47), *MiL* (40), *Cp* (30), *MiB* (28) and *Og* (22), suggesting best activity by *MiL.*

Antinociceptive studies revealed significant (*p* < 0.01) pain inhibition (%) as follows: Paracetamol (97), *Og* (113), *MiL* (108), *Pg* (84) and *MiB* (88). *Og* and *MiL* exhibited the best activities.

**Conclusion:**

The results obtained suggest that the constituent plants possess biologically active compounds with antipyretic, anti-inflammatory and antinociceptive activities. These activities are essential in the symptomatic treatment of malaria fever, thereby justifying the folk use of *Nefang*. This would be useful in its subsequent development for clinical application.

## Background

Overseas development assistance to low and middle income countries (LMICs) have led to substantial improvement in the number of vulnerable populations protected against malaria infection, especially in those who have access to drugs that effectively treat the disease [1]. In spite of this, *Plasmodium falciparum* malaria remains a major public health burden in most endemic African countries, where there is lack of access to modern healthcare facilities and disease monitoring is not well defined. The emerging resistance of the parasite to currently available drugs makes the situation even more complicated. The disease remains one of the major threats to public health and the economic development of these countries [2, 3]. Most of the populations resort to the use of plant-based complementary medicine for therapy [4].

Natural compounds have provided the best anti-malarials known to date. Examples include chloroquine from Cinchona species, artemisinin from *Artemisia annua* and atovaquone from *Tabebuia impetiginosa* [5, 6]. Their advantage for the development of drugs comes from the synergistic interactions of their components and their innate affinity for biological receptors [7]. The pharmacological justification of these principles could provide the basis for further development of plant-based traditional medicine as a reliable therapeutic tool.

Nefang is a polyherbal product composed of *Mangifera indica* (bark and leaf), *Psidium guajava, Carica papaya, Cymbopogon citratus, Citrus sinensis* and *Ocimum gratissimum* (leaves). It is frequently used for the treatment of malaria in the South West Region of Cameroon. Ethnopharmacological studies confirmed its formulation, folk use and a review of the biological activities of its constituent plants [8]. Use of Nefang for the treatment of malaria raises the question as to whether the therapeutic effects are as a result of the antiplasmodial activity of its constituents or to their ability to ameliorate the symptoms of malaria or a combination of both effects.

Evaluation of the in vitro and in vivo antioxidant properties of this polyherbal revealed potential free radical scavenging and antioxidant activities of some constituent plants. These activities correlated with their phenolic content and are believed to contribute to the demonstrated in vivo antioxidant activity of Nefang and play a role in curbing oxidative stress associated with malaria infection [9].

The in vitro antiplasmodial activities of this polyherbal and solvent extracts of its constituent plants has earlier been evaluated, revealing good activity and potential synergistic interactions between some of its components [10]. This synergy is believed to contribute favorably towards its antiplasmodial activity. The good antiplasmodial activity and demonstrated weak cytotoxicity of the constituent extracts suggested a high selectivity for *P. falciparum*. Preliminary phytochemical screening of its constituent plants revealed the presence of alkaloids, anthocyanins, flavonoids, phenols, saponins, tannins, triterpenes and sterols [10]. The synergy of these active and non-antiplasmodial compounds is responsible for the antiplasmodial activity of Nefang*.*

Our recent studies demonstrated the antimalarial efficacy of Nefang during early and established *Plasmodium* infection, revealing good suppression of parasitemia and chemotherapeutic activities [11]. We have also established the in vivo safety profile of Nefang.

This study aimed at providing pharmacological evidence on the folk use of Nefang by evaluating the effects of its constituent plants on induced pyrexia, inflammation and nociception in experimental rodent models.

## Methods

### Extraction of plant material

Fresh parts of the constituent plants of Nefang were identified and harvested from their natural habitat in Cameroon. Identification was done by a botanist, Dr. Tsabang Nole of the Institute of Medical Research and Medicinal Plants Studies (IMPM), Yaoundé, Cameroon with voucher specimen references as follows: *M. indica* (TN6225), *P. guajava* (TN6226), *C. papaya* (TN6227), *C. citratus* (TN6228), *C. sinensis* (TN6229), *O. gratissimum* (TN6230). Aqueous extractions were performed as earlier described [10] using pyrogen-free distilled water. Each filtrate was then concentrated in an air oven at 60 °C. The extracts were weighed and stored in labeled sealed plastic containers at 4 °C until use.

### Experimental animals

Swiss albino mice (20–25 g) were used for testing antinociceptive activity while wistar rats (170 – 200 g) were used for antipyretic and anti-inflammatory activities.

All experimental animals were housed under standard environmental conditions of temperature at 22-24 °C under a 12 h dark–light cycle, and allowed free access to drinking water and standard pellet diet.

Ethical approval for the study was obtained from Kenyatta National Hospital/University of Nairobi Ethics and Research committee, Nairobi-Kenya (KNH-ERC/A/324 - 5/12/12).

### Tests for antipyretic activity

#### D-Amphetamine induced pyrexia method

The antipyretic activities of the constituent aqueous plant extracts of Nefang were determined by the D-Amphetamine induced pyrexia method [12]. Wistar rats of both sexes were fasted for 24 h. At zero hour, the basal temperature of all the animals was taken using an infra-red thermometer. D-amphetamine (5 mgkg^−1^) was then administered to all the animals. After 30 min, sixty four animals with an increase in body temperature of 0.5 - 1 °C were selected for the study. They were randomized into sixteen groups of four rats each. Group 1 was treated with 10 mLkg^−1^ of the vehicle (normal saline), group 2 - the reference drug (Paracetamol, 150 mgkg^−1^) and two groups for each extract were treated orally with 200 and 400 mgkg^−1^ bwt of each extract. Body temperatures were obtained at 60, 120, 180 and 240 min after drug administration. The percentage suppression of induced pyrexia in the extract-treated groups was compared with that of the control to evaluate the activity of each extract.

#### Brewer’s yeast-induced hyperpyrexia method

Brewer’s yeast-induced hyperpyrexia method was used to determine the antipyretic activities of the constituent plants of Nefang [13]. Wistar rats of both sexes were fasted for 24 h and then randomized into sixteen groups of three rats each. At zero hour, the basal temperature of each rat was taken using an infra-red thermometer. Thereafter, each animal received a subcutaneous injection of 10 mLkg^−1^ of aqueous yeast suspension (20 % w/v) to elevate the body temperature. Eighteen hours post yeast injection, 10 mLkg^−1^ of the standard drug (aspirin, 100 mgkg^−1^), the vehicle (normal saline), 200 and 400 mgkg^−1^ of each aqueous extract were administered orally to different groups of rats. Body temperature of each animal was recorded at 0, 60, 120 and 180 min after drug administration and the activity of each extract evaluated.

### Test for anti-inflammatory activity

In vivo anti-inflammatory activities of the constituent plants of Nefang were evaluated using the carrageenan-induced rat paw edema method [14]. Ninety-two adult wistar rats were randomly divided into twenty three groups of four each. Paw volumes were measured at zero minute using the Archimedes principle of mercury displacement in a plethysmograph. Thereafter, 10 mLkg^−1^ of the vehicle (normal saline), the reference drug (Indomethacin, 10 mgkg^−1^), and 100, 200 and 400 mgkg^−1^ bwt of each extract were administered orally to different groups of rats. Thirty minutes later, paw edema was induced in each rat by injecting 0.1 mL of carrageenan (1 % in normal saline) to the right hind paw. Paw volumes were measured at 60, 120, 180 and 240 min. The difference between the paw volume at zero minute and each time point was taken as a measure of edema. The percentage inhibition of paw volumes in extract-treated groups was compared with the standard drug to evaluate the activity of the extracts.

### Tests for antinociceptive activity

#### Tail pressure method

The method described by Randall and Selitto [15] and modified by Kitchen [16] was used for the evaluation of analgesic activities of the constituent extracts. Thirty-six Swiss albino mice were randomly divided into nine groups of four each. At zero minute, the tail of each mouse was put on the tip of an analgesy-meter and pressure gradually increased up to a maximum of 25 units. The pressure at which the mouse began to struggle was noted and recorded. Mice in group 1 (positive control) were treated by intraperitoneal injection with 0.1 mL of the standard drug (morphine, 5 mgkg^−1^); group 2 – phosphate buffered saline (PBS); group 3 – 7 were each treated with 10 mLkg^−1^ of 1000 mgkg^−1^ bwt of each aqueous plant extract. After 30 min, mechanical pain was induced on the tail of each restrained mouse in turn by the use of analgesy-meter. The force was continuously monitored by a pointer moving along a linear scale. The weight causing pain before treatment (0 min) and then at 30, 60, 90 and 120 min post extract/drug treatment was recorded. Percentage pain inhibition (increase in pain threshold) produced relative to the control group was calculated for each extract-treated group and compared with the standard drug-treated group, using the following equation:

$$ \mathrm{Pain}\kern0.5em \mathrm{inhibition}\kern0.5em \left(\%\right)=\frac{\mathrm{Ft}\hbox{-} \mathrm{F}\mathrm{o}}{\mathrm{Fo}}\times 100 $$

Where Ft = Force at which the animal tries to free its tail after drug administration.

Fo = Force at which the animal tries to free its tail before drug administration.

Extracts that showed activity were then subjected to further dose–response testing.

#### Tail flick response method

The analgesic activity of each of the constituent plants of Nefang was determined by radiant heat tail-flick method [17]. The tail flick latency was assessed by an analgesiometer (Inco, India). Before selecting each mouse for the test, a baseline reaction was taken by placing the tip of the tail of each mouse on the radiant heat source from an analgesiometer. The strength of the current passing through the naked nichrome wire was kept constant at 5 amperes. The distance between the heat source and the mouse tails was 1.5 cm and the application site of the heat on the tail was maintained within 2 cm. Each mouse in turn was held in a suitable restrainer with tail protruding out. Radiant heat from the analgesiometer was applied on a designated spot on the tail. The time interval between the onset of stimulus and withdrawal (flicking) of the tail was taken as the reaction time for each mouse. Cut-off reaction time was 10 sec to avoid tissue injury during the process. The initial flicking time of each group was considered as the control reading.

One hundred and ten mice that responded positively were randomly divided into twenty-two groups of five each. Group 1 (positive control) was treated with 0.1 mL of the standard drug (Paracetamol, 10 mgkg^−1^) while the rest of the twenty-one groups were divided into three groups per aqueous extract and administered 300, 600 and 1200 mgkg^−1^ bwt respectively of each aqueous extract by intraperitoneal injection. The tip of the tail of each mouse was then exposed to the heat source from the analgesiometer and reaction time recorded at intervals of 30 min till 120 min. The percentage of pain inhibition was calculated for each extract-treated group and compared with that of the standard drug-treated group using the following equation:

$$ \mathrm{Percentage}\kern0.5em \mathrm{inhibition}=\frac{{\left(\mathrm{Test}\kern0.5em \mathrm{mean}\right)}_{\mathrm{t}}-{\left(\mathrm{Control}\kern0.5em \mathrm{mean}\right)}_{\mathrm{t}}}{{\left(\mathrm{Control}\kern0.5em \mathrm{mean}\right)}_{\mathrm{t}}}\times 100 $$

Where t = time

#### Hot plate method

The analgesic activity of each of the constituent plants of Nefang was tested using the hot plate method described by Janssen and Jagenea [18], with modifications. Ninety-two mice were randomly divided into twenty three groups of four mice each. Group 1 was treated with 0.1 mL of normal saline, group 2 - the reference drugs (piroxicam, 20 mgkg^−1^ and pethidine, 50 mgkg^−1^), while the remaining twenty one groups were divided into three groups per extract and treated with 300, 600 and 1200 mgkg^−1^ bwt of each extract respectively. An inverted 500 mL glass conical flask on a clamp and stand was connected by rubber tubing to a hot water bath with thermostat and pump, to enable free circulation of water. The temperature was regulated to 55 °C, such that the temperature on the surface of the inverted conical flask was the same as that of the water bath. Each mouse was then placed on the conical flask in order to obtain its response to heat-induced nociceptive pain stimulus. Licking of the forepaws and eventually jumping off the conical flask was taken as an indicator of the animal’s response to heat-induced nociceptive pain stimulus. The reaction time for each mouse was recorded in seconds. Readings were taken at time zero before administration of drugs/extracts and after that at intervals of 30, 60 and 120 min. Cut off time in the absence of response was 60 s to avoid tissue damage. The percentage of pain inhibition was calculated for each group using the following equation:

$$ \mathrm{Percentage}\kern0.5em \mathrm{inhibition}=\frac{{\left(\mathrm{Test}\kern0.5em \mathrm{mean}\right)}_{\mathrm{t}}\hbox{-} {\left(\mathrm{Control}\kern0.5em \mathrm{mean}\right)}_{\mathrm{t}}}{{\left(\mathrm{Control}\kern0.5em \mathrm{mean}\right)}_{\mathrm{t}}}\times 100 $$

Where t = time

### Statistical analysis

Data are expressed as mean ± standard deviation (SD). Data were analyzed using Windows SPSS Version 20.0 (SPSS Inc. Chicago, IL, USA). One-way analysis of variance (ANOVA) followed by Neuman-Keuls multiple comparison test to identify the differences between treated groups and controls. The data was considered significant at *P* < 0.05.

## Results

### Antipyretic activity

#### Effects on D-Amphetamine-induced pyrexia

Antipyretic activities of the constituent plant extracts of Nefang were evaluated using D-amphetamine induced pyrexia in experimental animals. The change in body temperature and the percentage inhibition of induced pyrexia by the constituent extracts have been summarized in Table 1. In rats treated with the standard drug, paracetamol (150 mgkg^−1^), we observed an initial rise in body temperature of approximately 1 °C after 30 min. The highest rise in body temperature observed after 60 min was less than 2 °C. However, after 120 min we observed a significant (p < 0.05) decrease in body temperature when compared to the control. This decrease to normal body temperature after 240 min, was significantly (*p* < 0.001) lower when compared to that of the control.

**Table 1:** Effect of the aqueous plant extracts of the constituents of *Nefang* on D-amphetamine-induced pyrexia in wistar rats

Treatment (Drug/Aqueous Extract)	Dose (mg/kg)	Basal Temperature	Body temperature [BT] (°C) and pyrexia inhibition [PI] (%) over time (minutes) $\left(\overline{\mathrm{x}}\pm \mathrm{S}\mathrm{D},\kern0.5em \mathrm{n}=4\right)$									
			30		60		120		180		240	
			BT	PI	BT	PI	BT	PI	BT	PI	BT	PI
Normal Saline	-	34.95 ± 0.33	35.78 ± 0.19	0.0	36.23 ± 0.32	0.0	36.70 ± 0.38	0.0	36.78 ± 0.39	0.0	36.98 ± 0.33	0.0
Paracetamol	150	34.98 ± 0.42	35.85 ± 0.24	15.5	36.18 ± 0.28	15.9	36.08 ± 0.19*	37.1	35.28 ± 0.29*	78.1	34.98 ± 0.17**	100.0
*Mangifera indica* bark	200	35.03 ± 0.10	36.40 ± 0.14	0.0	35.90 ± 0.22	31.4	35.60 ± 0.16*	67.1	35.45 ± 0.13*	76.7	35.25 ± 0.33*	88.9
	400	35.00 ± 0.08	36.55 ± 0.21	0.0	36.03 ± 0.15	19.6	35.68 ± 0.22*	61.4	35.45 ± 0.17*	75.3	35.33 ± 0.24*	84.0
*Mangifera indica* leaf	200	35.00 ± 0.18	36.05 ± 0.13	0.0	35.98 ± 0.19	23.5	35.75 ± 0.29*	57.1	35.43 ± 0.17*	76.7	35.25 ± 0.26*	87.7
	400	35.18 ± 0.34	36.80 ± 0.24	0.0	36.78 ± 0.10	0.0	36.08 ± 0.10*	48.6	35.85 ± 0.45*	63.0	35.38 ± 0.37*	90.1
*Psidium guajava* leaf	200	35.18 ± 0.39	36.13 ± 0.26	0.0	36.35 ± 0.37	7.8	36.80 ± 0.14	7.1	36.63 ± 0.19	20.5	36.45 ± 0.06	37.0
	400	35.15 ± 0.21	36.05 ± 0.19	0.0	36.50 ± 0.54	0.0	36.68 ± 0.13	12.9	36.40 ± 0.14	31.5	36.45 ± 0.33	35.8
*Carica papaya leaf*	200	35.05 ± 0.48	36.48 ± 0.61	0.0	36.53 ± 0.43	0.0	36.78 ± 0.49	1.4	36.80 ± 0.48	4.1	36.68 ± 0.39	19.8
	400	35.00 ± 0.35	36.45 ± 0.52	0.0	36.63 ± 0.93	0.0	36.70 ± 0.22	2.9	36.75 ± 0.52	4.1	36.75 ± 0.37	13.6
*Cymbopogon citratus leaf*	200	35.05 ± 0.17	36.10 ± 0.08	0.0	35.88 ± 0.22	35.3	35.50 ± 0.38*	74.3	35.20 ± 0.41*	91.8	35.15 ± 0.17**	95.1
	400	34.90 ± 0.29	35.85 ± 0.06	0.0	35.75 ± 0.25	33.3	35.43 ± 0.33*	70.0	35.23 ± 0.42*	82.2	35.20 ± 0.18**	85.2
*Citrus sinensis leaf*	200	34.90 ± 0.08	35.73 ± 0.13	0.0	36.05 ± 0.13	9.8	35.73 ± 0.15*	52.9	35.50 ± 0.12*	67.1	35.28 ± 0.22**	81.5
	400	34.98 ± 0.15	35.93 ± 0.10	0.0	36.08 ± 0.28	13.7	35.88 ± 0.15*	48.6	35.58 ± 0.32*	67.1	35.33 ± 0.22**	82.7
*Ocinum gratissimum leaf*	200	34.93 ± 0.13	35.63 ± 0.36	15.1	35.80 ± 0.36	31.4	35.58 ± 0.36*	62.9	35.28 ± 0.66*	80.8	35.28 ± 0.71**	82.7
	400	34.90 ± 0.16	35.95 ± 0.17	0.0	36.05 ± 0.17	9.8	35.88 ± 0.13*	44.3	35.25 ± 0.32*	80.8	35.20 ± 0.22**	85.2

Siginificant difference * - *p* < 0.05. ** - *p* < 0.001 when compare to the control

Upon administration of the constituent plant extracts of *Nefang*, we observed the same rise in body temperature after 30 min in all groups, with highest body temperatures attained after about 60 min. After 120 min, we observed significant (*p* < 0.05) decrease in body temperatures in animals treated with *Cc*, *Og*, *MiL*, *MiB* and *Cs*. After 240 min, we observed that *Cc* exhibited the best activity with pyrexia inhibition of 95 %, followed by *MiL* (90), *MiB* (88), *Og* (85) and *Cs* (82), comparable to the standard drug, paracetamol (100). *Pg* and *Cp* exhibited very weak antipyretic activities in experimental animals.

#### Effects on brewer’s yeast-induced hyperpyrexia

Antipyretic activities of the constituent plant extracts of Nefang were further evaluated using brewer’s yeast-induced hyperpyrexia in experimental animals. The effect on the body temperatures and the percentage inhibition of induced pyrexia by the constituent plant extracts have been summarized in Table 2. In rats treated with the standard drug, paracetamol (150 mgkg^−1^), we observed the same trend in temperature rise like the previous exercise with D-amphetamine, with body temperatures of experimental animals coming back to normal after 240 min and significantly (*p* < 0.001) lower when compared to that of the control.

**Table 2:** Effect of the aqueous extracts of the constituent plants of *Nefang* on Brewer’s Yeast-induced hyperpyrexia in wistar rats

Treatment (Drug/Aqueous Extract)	Dose (mg/kg)	Basal Temperature	Body temperature [BT] (°C) and pyrexia inhibition [PI] (%) over time (minutes) $\left(\overline{\mathrm{x}}\pm \mathrm{S}\mathrm{D},\kern0.5em \mathrm{n}=4\right)$									
			30		60		120		180		240	
			BT	PI	BT	PI	BT	PI	BT	PI	BT	PI
Normal Saline	-	37.00 ± 0.29	37.95 ± 0.21	0.0	38.14 ± 0.38	0.0	38.65 ± 0.32	0.0	38.91 ± 0.11	0.0	38.96 ± 0.54	0
Paracetamol	150	36.90 ± 0.38	37.70 ± 0.45	15.8	37.75 ± 0.33	25.4	37.85 ± 0.28*	42.4	37.47 ± 0.26*	70.2	37.00 ± 0.39**	94.9
*Mangifera indica* bark	200	36.90 ± 0.31	37.85 ± 0.39	0.0	37.90 ± 0.41	12.3	37.75 ± 0.27*	48.5	37.55 ± 0.25*	66.0	37.20 ± 0.41*	84.7
	400	36.95 ± 0.92	37.95 ± 0.27	0.0	38.15 ± 0.54	0.0	37.73 ± 0.31*	52.7	37.50 ± 0.19*	71.2	37.30 ± 0.38*	82.1
*Mangifera indica* leaf	200	37.00 ± 0.74	37.85 ± 0.16	10.5	37.95 ± 0.37	16.7	37.60 ± 0.18*	63.6	37.35 ± 0.11*	81.7	37.20 ± 0.28*	89.8
	400	37.05 ± 0.67	38.45 ± 0.43	0.0	38.60 ± 0.29	0.0	38.25 ± 0.17*	27.3	37.90 ± 0.28*	55.5	37.50 ± 0.19*	77.0
*Psidium guajava* leaf	200	36.95 ± 0.51	38.00 ± 0.54	0.0	38.40 ± 0.61	0.0	38.70 ± 0.19	0.0	38.65 ± 0.38	11.0	38.50 ± 0.17	20.9
	400	37.10 ± 0.33	38.00 ± 0.15	5.3	38.65 ± 0.72	0.0	38.75 ± 0.24	0.0	38.55 ± 0.36	24.1	38.45 ± 0.11	31.1
*Carica papaya leaf*	200	37.00 ± 0.61	38.25 ± 0.27	0.0	38.60 ± 0.36	0.0	38.68 ± 0.22	0.0	38.65 ± 0.31	13.6	38.60 ± 0.26	18.4
	400	36.90 ± 0.68	37.90 ± 0.51	0.0	38.45 ± 0.29	0.0	38.65 ± 0.31	0.0	38.70 ± 0.29	5.8	38.60	13.3
*Cymbopogon citratus leaf*	200	36.95 ± 0.28	37.90 ± 0.89	0.0	37.85 ± 0.18	21.1	37.45 ± 0.35*	69.7	37.20 ± 0.52*	86.9	37.00 ± 0.09**	97.4
	400	37.00 ± 0.41	38.05 ± 0.62	0.0	37.70 ± 0.09	38.6	37.45 ± 0.18*	72.7	37.30 ± 0.56*	84.3	37.05 ± 0.22**	97.4
*Citrus sinensis leaf*	200	37.00 ± 0.22	38.00 ± 0.20	0.0	38.05 ± 0.25	7.9	37.85 ± 0.26*	48.5	37.55 ± 0.19*	71.2	37.35 ± 0.35*	82.1
	400	37.05 ± 0.15	38.05 ± 0.31	0.0	38.25 ± 0.18	0.0	37.75 ± 0.11*	57.6	37.45 ± 0.55*	79.1	37.25 ± 0.28*	89.8
*Ocimumgratissimum leaf*	200	37.00 ± 0.33	37.95 ± 0.46	0.0	37.90 ± 0.42	21.1	37.75 ± 0.19*	54.5	37.40 ± 0.38*	79.1	37.15 ± 0.52**	92.3
	400	37.00 ± 0.81	37.95 ± 0.19	0.0	38.00 ± 0.19	12.3	37.75 ± 0.09*	54.5	37.45 ± 0.61*	76.4	37.10 ± 0.33**	94.9

Significant difference * - *p* < 0.05, ** - *p* < 0.001 when compared to the control

*Cc*, *Og*, *MiL*, *MiB* and *Cs* exhibited significant (*p* < 0.05) better antipyretic activities while *Pg* and *Cp* exhibited lower activities when compared to the control. At the end of 240 min, *Cc* and *Og* exhibited percentage pyrexia inhibition of 97 and 94, comparable to the activity of the standard drug paracetamol (95). We observed once again that, *Cc* exhibited the best activity among all the extracts tested. We also observed that pyrexia inhibition trends by the constituent plant extracts and the standards were comparable in both methods.

### Anti-inflammatory activity

The anti-inflammatory activities of the constituent plant extracts of Nefang at different concentrations (100, 200 and 400 mgkg^−1^) were determined using Indomethacin (10 mgkg^−1^) as the standard. Results obtained have been summarized in Table 3. These indicated significant inhibition of carrageenan-induced inflammation in paws of experimental rats when compared to the control. Inflammation of the paw of experimental animals was observed 60 min post carrageenan administration. In experimental rats treated with the standard drug, the paw volumes were significantly (*p* < 0.001) reduced, 240 min post administration, giving a paw edema inhibition of 43.7 % when compared to the control. After administration of the constituent plant extracts of Nefang, only *MiL* exhibited a comparable anti-inflammatory activity to that of the standard drug by significantly (*p* < 0.001) reducing the inflamed rat paw volumes by 40 % at all doses. *Cp* and *MiB* also exhibited significant (*p* < 0.05) anti-inflammatory activities at lower doses in experimental rats when compared to the control, with approximately 28 – 30 % paw inhibition. *Og*, *Pg*, *Cc* and *Cs* all showed moderate but significant (*p* < 0.05) anti-inflammatory activities at lower doses, with paw inhibition of approximately 20 %.

**Table 3:** Anti-inflammatory effect of the constituent plant extracts of *Nefang* on carrangeenan-induced paw edema in wistar rats

Treatment (Drug/Aqueous Extract)	Dose (mg/kg)	Increase in paw volumes [PV] (mL) and edema inhibition [EI] (%) over time (minutes) $\left(\overline{\mathrm{x}}\pm \mathrm{S}\mathrm{D},\kern0.5em \mathrm{n}=5\right)$								
		0	60		120		180		240	
			PV	EI	PV	EI	PV	EI	PV	EI
Normal Saline	-	0	1.18 ± 0.08	0.0	1.28 ± 0.14	0.0	1.46 ± 0.13	0.0	1.56 ± 0.09	0.0
Indomethacin	10	0	1.17 ± 0.04	1.3	1.08 ± 0.03*	15.3	1.00 ± 0.10**	31.5	0.88 ± 0.03**	43.7
*Mangifera indica* bark	100	0	1.07 ± 0.11	9.7	1.13 ± 0.09*	11.4	1.19 ± 0.03*	18.8	1.12 ± 0.01**	28.3
	200	0	1.15 ± 0.12	3.0	1.21 ± 0.02	5.5	1.32 ± 0.06*	9.9	1.24 ± 0.10*	20.6
	400	0	1.18 ± 0.05	0.4	1.27 ± 0.04	0.8	1.36 ± 0.03	6.8	1.26 ± 0.06*	19.0
*Mangifera indica* leaf	100	0	1.04 ± 0.13	11.9	1.01 ± 0.08*	20.8	0.92 ± 0.08**	37.3	0.93 ± 0.06**	40.2
	200	0	1.05 ± 0.03	11.4	1.11 ± 0.08*	13.3	0.91 ± 0.14**	37.7	0.95 ± 0.11**	39.2
	400	0	1.05 ± 0.03	11.4	0.93 ± 0.03*	27.5	0.89 ± 0.06**	39.4	0.88 ± 0.08**	43.4
*Psidium guajava* leaf	100	0	1.04 ± 0.04	11.9	1.12 ± 0.10*	12.5	1.12 ± 0.06*	23.6	1.21 ± 0.04*	22.2
	200	0	1.04 ± 0.07	11.9	1.14 ± 0.11*	11.0	1.13 ± 0.13*	22.6	1.23 ± 0.13*	21.2
	400	0	1.08 ± 0.07	8.5	1.17 ± 0.10*	8.2	1.27 ± 0.15*	13.0	1.28 ± 0.05*	17.7
*Carica papaya leaf*	100	0	0.97 ± 0.03	17.8	1.04 ± 0.10*	18.4	1.15 ± 0.09**	21.2	1.10 ± 0.03**	29.3
	200	0	1.10 ± 0.09	6.8	1.12 ± 0.17*	12.5	1.17 ± 0.13**	19.9	1.11 ± 0.05**	28.9
	400	0	1.11 ± 0.07	6.4	1.19 ± 0.05	6.7	1.36 ± 0.11	7.2	1.32 ± 0.09*	15.1
*Cymbopogon citratus leaf*	100	0	1.08 ± 0.12	8.9	1.16 ± 0.04*	9.4	1.24 ± 0.11*	15.1	1.27 ± 0.03*	18.3
	200	0	1.12 ± 0.04	5.1	1.17 ± 0.08*	8.2	1.20 ± 0.02*	18.2	1.34 ± 0.04*	13.8
	400	0	1.15 ± 0.04	3.0	1.24 ± 0.01	3.1	1.42 ± 0.06	3.1	1.48 ± 0.09	5.1
*Citrus sinensis leaf*	100	0	1.15 ± 0.12	2.5	1.22 ± 0.03	4.7	1.24 ± 0.05*	15.1	1.27 ± 0.13*	18.6
	200	0	1.17 ± 0.03	1.3	1.19 ± 0.04	7.1	1.35 ± 0.20	7.5	1.46 ± 0.16	6.4
	400	0	1.17 ± 0.04	0.8	1.20 ± 0.03	6.3	1.45 ± 0.04	1.0	1.56 ± 0.13	0.0
*Ocimum gratissimum leaf*	100	0	1.06 ± 0.05	10.6	1.14 ± 0.07*	10.6	1.23 ± 0.04*	16.1	1.22 ± 0.05*	21.5
	200	0	1.07 ± 0.06	9.3	1.16 ± 0.08*	9.4	1.23 ± 0.04*	16.1	1.24 ± 0.02*	20.3
	400	0	1.17 ± 0.03	1.3	1.26 ± 0.04	1.2	1.31 ± 0.12	10.3	1.33 ± 0.08	14.5

Significant difference: compared to control; (*)-*p* < 0.05, (**)-*p* < 0.01

### Antinociceptive activity

#### Effects on tail pressure

**Fig. 1:**
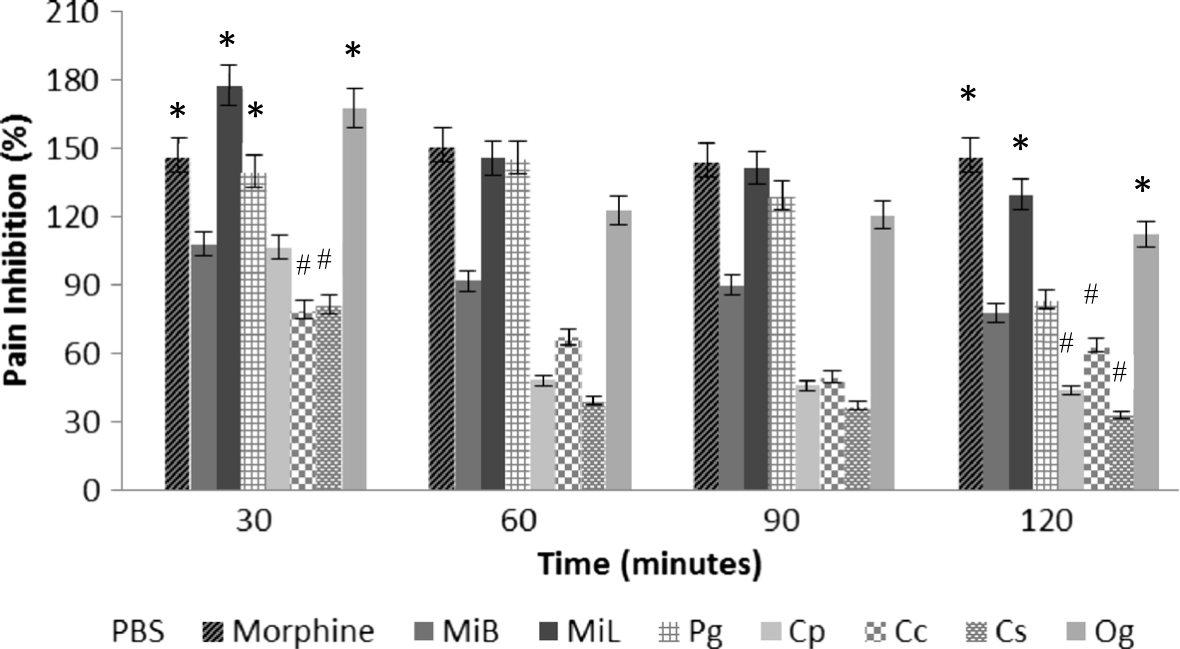
Antinociceptive effect of the constituent plant extracts of *Nefang* on tail-induced pressure in mice. Significant difference: * - *p* < 0.001 when compared to normal control; # - *p* < 0.05 when compared to the standard

The antinociceptive activity of the constituent plant extracts of Nefang on tail-induced pressure in mice was determined using an analgesy-meter. The percentage inhibition of induced pain in experimental mice, calculated from their mean reaction times to gradual increase in tail pressure has been summarized in Fig. 1. The results obtained suggested that *MiL*, *Og* and *Pg* produced significant (*p* < 0.001) percentage inhibition of the threshold pain by 177.3, 167.4 and 139.6 respectively, when compared to the normal control 30 min after extract administration. This activity was comparable to that of the standard drug, morphine (146.7 %). *MiB* and *Cp* had moderate percentage pain inhibition of 107.7 and 106.3 respectively while *Cs* and *Cc* had weak activities. Two hours post treatment, only *MiL* and *Og* still exhibited good pain inhibition levels of 129.6 % and 112.2 % respectively, comparable to 146.7 % for morphine. *Pg* and *MiB* still exhibited moderate activities at 83.3 % and 77.4 %, while *Cc*, *Cp* and *Cs* exhibited very weak activities. Since all the extracts showed activity, they were all subjected to further dose–response testing.

#### Tail flick response

The results of the tail flick response in mice and the calculated percentage inhibition by the constituent plant extracts of Nefang have been presented in Table 4. In experimental animals treated with the standard drug, paracetamol, the mean initial tail flicking time was 4.27 s. The reaction time increased gradually after administration of the standard drug and a mean reaction time of 8.39 s was attained at the end of 60 min. This was constant up to the end of 120 min. Analysis revealed that *Og*, *MiL*, *Pg* and *MiB* exhibited dose-dependent increases in reaction time. The reaction times after administration of *MiL* and *Pg* at doses of 600 and 1200 mgkg^−1^ bwt, were comparable to that of the standard group after 60 min and up to 120 min, whereas animals treated with *Og* and *MiB* exhibited the same activity only at the highest dose of 1200 mgkg^−1^. The mean initial tail flicking time resulted in a pain inhibition of 59.9 % after 30 min to over 90 % after 60 min, which was constant to the end of the experiment (120 min), when compared to the reaction before drug administration. Pain inhibition (%) at the highest doses for *Og* (113), *MiL* (108), *Pg* (84) and *MiB* (88) were comparable to that of paracetamol (97). At lower doses, we observed significantly (*p* < 0.05) lower activities of these extracts when compared to the standard. In animals administered *Cp* and *Cc*, we observed moderate reaction times at doses up to 600 mgkg^−1^ bwt, though significantly (*p* < 0.05) low when compared to the standard. At higher doses as well as in animals treated with *Cs* (all doses), reaction times were significantly (*p* < 0.001) low, suggesting that they exhibited lower activities.

**Table 4:** Antinociceptive effect of the constituent plant extracts of *Nefang* on electric heat-induced pain on the tails of mice

Treatment (Drug/Aqueous Extract)	Dose (mg/kg)	Tail flick reaction time [RT] (sec) and pain inhibition [PI] (%) at designated time intervals (minutes) (x ± SD, n = 5)								
		0	30		60		90		120	
		RT	RT	PI	RT	PI	RT	PI	RT	PI
Paracetamol	150	4.27 ± 0.38	6.82 ± 0.28	60.0	8.39 ± 0.20	96.8	8.43 ± 0.36	97.6	8.26 ± 0.23	93.5
*Mangifera indica* bark	300	4.30 ± 0.38	5.62 ± 0.24	31.7	6.47 ± 0.37	51.7	6.30 ± 0.31*	47.6	5.66 ± 0.24*	32.7
	600	4.30 ± 0.38	5.96 ± 0.49	39.6	6.46 ± 0.37*	51.5	6.35 ± 0.25*	48.8	6.25 ± 0.20*	46.5
	1200	4.30 ± 0.38	7.20 ± 0.27	68.8	8.05 ± 0.41	88.7	6.99 ± 0.29	63.8	6.98 ± 0.06	63.6
*Mangifera indica* leaf	300	4.32 ± 0.38	6.47 ± 0.25	51.8	7.59 ± 0.43	77.9	6.19 ± 0.37*	45.1	6.08 ± 0.20*	42.6
	600	4.32 ± 0.38	6.95 ± 0.14	62.9	7.80 ± 0.21	82.2	7.78 ± 0.76	82.4	7.47	75.1
	1200	4.32 ± 0.38	7.09 ± 0.48	66.1	8.89 ± 0.41	108.5	8.64 ± 0.44	102.6	8.42 ± 0.36	97.3
*Psidium guajava* leaf	300	4.25 ± 0.38	6.32 ± 0.35	49.3	6.49 ± 0.29	52.2	6.38 ± 0.17*	49.6	5.26 ± 0.17*	23.2
	600	4.25 ± 0.38	7.63 ± 0.38	78.8	7.73 ± 0.27	81.2	7.42 ± 0.46	73.8	6.45 ± 0.31*	51.2
	1200	4.25 ± 0.38	7.82 ± 0.35	83.3	7.88 ± 0.19	84.7	7.54 ± 0.37	76.7	7.08 ± 0.05	65.9
*Carica papaya leaf*	300	4.29 ± 0.38	5.80 ± 0.70*	36.1	6.35 ± 0.31*	48.9	6.19 ± 0.53*	45.1	5.98 ± 0.17*	40.2
	600	4.29 ± 0.38	6.73 ± 0.87	57.8	6.65 ± 0.52	55.8	6.34 ± 0.48*	48.7	6.16 ± 0.27*	44.4
	1200	4.29 ± 0.38	5.04 ± 0.62*	18.2	5.12 ± 0.39*	20.1	5.39 ± 0.63*	26.3	5.36 ± 0.29*	25.7
*Cymbopogon citratus leaf*	300	4.31 ± 0.38	5.42 ± 0.50*	27.0	6.22 ± 0.28*	45.8	5.48 ± 0.09	28.4	5.00 ± 0.38*	17.3
	600	4.31 ± 0.38	6.00 ± 0.90	40.6	6.43 ± 0.33	50.8	5.98 ± 0.30*	40.1	5.74 ± 0.38*	34.5
	1200	4.31 ± 0.38	5.87 ± 0.32	37.6	6.56 ± 0.41	53.7	5.80 ± 0.25*	36.0	5.69 ± 0.32*	33.4
*Citrus sinensis leaf*	300	4.27 ± 0.38	4.76 ± 0.17*	11.6	4.77 ± 0.31**	11.7	4.61 ± 0.32**	8.0	4.30 ± 0.22**	0.9
	600	4.27 ± 0.38	4.92 ± 0.28*	15.4	4.59 ± 0.33**	7.5	4.65 ± 0.42**	9.1	4.35 ± 0.15**	1.9
	1200	4.27 ± 0.38	4.55 ± 0.30*	6.7	4.35 ± 0.24**	1.9	4.38 ± 0.15**	2.8	4.26 ± 0.22**	0.0
*Ocimum gratissimum leaf*	300	4.33 ± 0.38	5.58 ± 0.38	30.8	6.33 ± 0.50*	48.5	6.45 ± 0.26*	51.2	5.85 ± 0.14*	37.0
	600	4.33 ± 0.38	6.03 ± 0.76	41.3	6.60 ± 0.54	54.7	7.09 ± 0.68	66.1	6.19 ± 0.30*	45.1
	1200	4.33 ± 0.38	7.50 ± 0.55	75.9	7.81 ± 0.29	83.0	9.09 ± 0.69	113.0	7.91 ± 0.38	85.5

Significant difference * - *p* < 0.05, ** - *p* < 0.001 when compared to the standard control

#### Hot plate response

The effect of the constituent plants of Nefang on heat-induced nociceptive pain in mice was determined using the hot plate method (Table 5). Piroxicam (20 mgkg^−1^) and pethidine (50 mgkg^−1^) were used as standards. The reaction times of experimental animals were taken after 30, 60 and 120 min. Piroxicam-treated rats exhibited reactions times of 19.31, 47.49 and 47.41 s and pethidine-treated rats, 18.90, 19.15 and 20.02 s. These produced significant percentage increase in the threshold of pain by 87.7, 361.6, 360.8 (*p* < 0.01) and 100.2, 102.8, 112.0 (*p* < 0.05) respectively, suggesting that piroxicam exhibited a better antinociceptive activity than pethidine relative to the control.

**Table 5:** Antinociceptive effect of the constituent plant extracts of *Nefang* on heat-induced nociceptive pain in mice

Treatment (Drug/Aqueous Extract)	Dose (mg/kg)	Mice reaction time [RT] (sec) and pain inhibition [PI] (%) at designated time intervals (minutes) $\left(\overline{\mathrm{x}}\pm \mathrm{S}\mathrm{D},\kern0.5em \mathrm{n}=4\right)$							
		0 min		30 min		60 min		120 min	
		RT	PI	RT	PI	RT	PI	RT	PI
Normal Saline	-	9.51 ± 1.24	0	10.70 ± 1.07	12.6	10.56 ± 0.74	11.0	11.28 ± 0.67	18.6
Piroxicam	20	10.29 ± 0.61	0	19.31 ± 1.34*	87.7	47.49 ± 0.76**	361.6	47.41 ± 0.66**	360.8
Pethidine	50	9.44 ± 0.17	0	18.90 ± 0.92*	100.2	19.15 ± 0.77*	102.8	20.02 ± 1.14*	112.0
*Mangifera indica* bark	300	9.93 ± 0.25	0	13.90 ± 1.21	40.0	21.75 ± 1.28*	119.0	20.81 ± 0.68*	109.6
	600	9.63 ± 0.69	0	13.65 ± 0.34	41.8	22.23 ± 0.71*	131.0	22.61 ± 0.70*	134.8
	1200	10.05 ± 0.26	0	22.91 ± 0.89*	128.0	27.29 ± 0.62*	171.7	23.79 ± 1.81*	136.8
*Mangifera indica* leaf	300	11.11 ± 0.94	0	12.80 ± 0.58	15.2	16.71 ± 0.50*	50.4	16.11 ± 0.26	45.0
	600	11.21 ± 0.48	0	23.72 ± 0.88*	111.6	24.25 ± 1.17*	116.3	21.71 ± 0.68*	93.7
	1200	10.70 ± 0.53	0	23.41 ± 1.07*	118.8	32.75 ± 0.25**	206.1	28.56 ± 0.13*	167.0
*Psidium guajava* leaf	300	10.09 ± 0.45	0	14.41 ± 0.73	42.8	14.57 ± 0.68	44.4	13.79 ± 1.14	36.6
	600	9.84 ± 0.34	0	14.86 ± 0.51	51.1	15.47 ± 0.21	57.3	18.33 ± 0.29	86.3
	1200	10.29 ± 0.53	0	21.27 ± 0.46*	106.7	26.29 ± 0.24*	155.4	24.22 ± 0.57*	135.3
*Carica papaya leaf*	300	9.63 ± 0.48	0	11.46 ± 0.94	19.0	11.51 ± 0.36	19.5	10.79 ± 0.61	12.0
	600	9.45 ± 0.40	0	12.28 ± 0.84	30.0	12.51 ± 0.38	32.5	11.01 ± 0.72	16.6
	1200	9.90 ± 0.77	0	12.45 ± 0.28	25.8	17.42 ± 0.25*	76.0	16.91 ± 0.62	70.8
*Cymbopogon citratus leaf*	300	10.07 ± 0.34	0	11.00 ± 0.36	9.2	11.71 ± 0.38	16.3	15.28 ± 0.56	51.7
	600	9.78 ± 0.34	0	13.34 ± 0.77	36.4	13.02 ± 0.45	33.1	16.08 ± 0.72	64.4
	1200	10.05 ± 0.33	0	15.24 ± 1.12	51.6	14.81 ± 1.10	47.3	18.85 ± 0.57	75.0
*Citrus sinensis leaf*	300	9.90 ± 0.44	0	13.24 ± 0.60	33.7	12.83 ± 0.24	29.6	13.27 ± 0.70	34.1
	600	9.86 ± 0.46	0	15.10 ± 0.61	53.2	14.99 ± 0.90	52.1	13.60 ± 0.43	38.0±
	1200	9.73 ± 0.33	0	14.21 ± 0.67	46.0	16.34 ± 0.60*	67.9	15.03 ± 1.37	54.5
*Ocimum gratissimum leaf*	300	9.63 ± 0.36	0	13.66 ± 0.30	41.8	18.15 ± 0.89*	88.5	18.98 ± 0.92	97.0
	600	9.79 ± 0.28	0	13.66 ± 0.89	39.5	22.83 ± 0.20*	133.2	24.29 ± 1.17*	148.1
	1200	10.07 ± 0.70	0	28.98 ± 0.64*	187.7	29.94 ± 0.43**	197.2	32.55 ± 1.47**	223.1

Significant difference * - *p* < 0.05; ** - *p* < 0.001 when compared to the control

After administration of the plants extracts, we observed a dose-dependent increase in reaction times in animals treated with *Og*, *MiL*, *MiB* and *Pg* between 30 to 120 min, with *Og* showing the best reaction time of 28.98, 29.94 and 32.55 s after 30, 60 and 120 s respectively, at the highest dose of 1200 mgkg^−1^. This corresponds to an increased percentage pain inhibition of 187.7, 197.2 and 223.1 during the experimental time; this was significantly (*p* < 0.001) lower than that of piroxicam after 120 min and higher than that of pethidine (*p* < 0.001). *MiL*, *MiB* and *Pg* all showed significantly (*p* < 0.001) higher pain inhibition at 1200 mgkg^−1^ in experimental animals when compared to animals that were treated with pethidine, though lower when compared to animals that were administered piroxicam. *Cp*, *Cc* and *Cs* exhibited weak activities (*p* < 0.001).

## Discussion

Infections such as malaria, caused by *Plasmodium* species usually trigger a cascade of unpleasant physiological changes in the host such as oxidative stress, pyrexia, inflammation and nociception. Biological agents that can act against these and other relevant effects can complement therapeutic activities of antiparasitic and antimicrobial agents. Activities evaluated in the constituent plants of *Nefang* included antipyretic, anti-inflammatory and antinociceptive.

Pyrexia is a result of the secondary impact of infection, tissue damage, inflammation, graft rejection, malignancy or other diseased states including malaria. Brewer’s yeast and D-amphetamine are commonly used to induce pyrexia in rats and mice [19]. Brewer’s yeast induces pyrexia by increasing the synthesis of prostaglandins (PGE2). The infections or damaged tissue serves as a pyrogenic stimulus and the pyrogens are phagocytized by the Kupffer cells, monocytes and macrophages leading to the release of pro-inflammatory mediators (cytokines). These cause an increase in the synthesis of prostaglandins near pre-optic hypothalamus area thereby triggering the hypothalamus to elevate the set point of normal body temperature [20]. Amphetamine on the other hand, acts on the brain causing the release of biogenic amines from their storage sites in nerve terminals. This results in increased level of cyclic-Adenosine monophosphate (cAMP) and subsequent synthesis of prostaglandins from arachidonic acids produced in neurons receptor-mediated hydrolysis of phospholipids, leading to hyperthermia [21]. Most of the antipyretic drugs inhibit cyclooxygenase (COX-2) expression thereby inhibiting prostaglandin biosynthesis and reducing elevated body temperature. They are, however, toxic to the hepatic cells, glomeruli, cortex of the brain and heart muscle. Natural antipyretic remedies with minimal toxicity are therefore essential.

The results of this study show that *Cc*, *Og*, *MiL*, *MiB* and *Cs* aqueous extracts significantly reduced amphetamine and brewer’s yeast induced hyperthermia in the rats at 120, 180 and 240 min post administration. This hypothermic activity might have been achieved by their action on COX-2, thereby reducing the concentration of prostaglandin in the brain or by enhancing the inherent production of the body’s own antipyretic substances such as arginine and vasopressin [22]. An alternative could have been by vasodilation of superficial blood vessels leading to increased dissipation of heat as a result of a reset of the hypothalamic temperature control centre [23].

Inflammation is a common phenomenon in malaria infection and it is the reaction of living tissues towards injury. The carrageenan-induced paw edema is a prototype for the exudative phase of acute inflammatory effects. The development of edema in the rat paw after the injection of carrageenan has been described as a biphasic event [24]. The initial phase which starts immediately after injection and reduces within one hour, is attributed to the release of histamine and serotonin, while the second phase of swelling which begins at one and remains through three hours, is due to the release of prostaglandin-like substances [25]. The second phase of edema is sensitive to both clinically useful steroidal and non-steroidal anti-inflammatory drugs (NSAIDs). Generally NSAIDs strongly inhibit the second phase of carrageenan-induced edema. The significant inhibition of paw edema in rats by *MiL*, *Cp* and *MiB* aqueous extracts suggest that they may contain biologically active substances with anti-inflammatory properties. The significant anti-inflammatory activities exhibited may be due to the inhibition of any inflammatory mediators, and this might contribute greatly to the antimalarial activity of Nefang. The co-existence of antinociceptive and anti-inflammatory activities observed in Nefang, are properties shared by most NSAIDs, particularly the salicylates and their derivatives. The therapeutic benefits of traditional remedies are often attributed to a combination of active constituents [26].

Increased nociception is a common phenomenon during *Plasmodium* infection. Measuring antinociceptive activity is based on the principle that inflammation increases the sensitivity to pain and that this sensitivity is susceptible to modification by analgesics. Inflammation decreases the pain reaction threshold and this low pain reaction threshold is readily elevated by different types of analgesics [15]. Antinociceptive properties were studied using models that could provide different types of noxious stimuli. These were mechanical pressure on the tail, thermal stimuli using the radiant heat-induced nociceptive pain on the tail and heat-induced nociceptive pain on the paws of mice. Mechanical pain induced by the analgesy-meter provides a model for the study of non-inflammatory pain. The analgesy-meter antinociceptive test is useful in elucidating centrally mediated antinociceptive responses, which focuses mainly on changes above the spinal cord level [27]. The significant increase in pain threshold produced by the extracts in the analgesy-meter test suggests involvement of central pain pathways. Thermal tests have several advantages including the sensitivity to strong analgesics and limited tissue damage [28]. Furthermore, they utilize phasic stimulus of high intensities mimicking responses in conditions that involve high threshold pain of short duration. However, a disadvantage of this model is that since it is short lasting, it does not assess modulatory mechanisms that may be triggered by the stimulus itself [29] and is a less valid model for clinical pain [30]. The results obtained indicate that *Og*, *MiL*, *Pg* and *MiB* can significantly inhibit responses to mechanical and thermal stimuli. The inhibition of thermal stimuli was dose dependent, thus indicating that these extracts, at the doses administered, had strong antinociceptive activities and can contribute significantly to the antimalarial effect of Nefang.

The results obtained in these studies are consistent with previously reports on the pharmacological activities of *O. gratissimum* [31], *M. indica* bark and leaf [32, 33], *C. papaya* [34] and *P. guajava* [35]. These findings lend pharmacological support to the reported folkloric uses of Nefang in the treatment of malaria and the role played by each of them.

## Conclusion

The results obtained suggest that the constituent plants possess biologically active compounds with antipyretic, anti-inflammatory and antinociceptive activities. These findings define the role of each constituent plant, suggesting that these pharmacological activities are essential in the symptomatic management of malaria fever. These also provide scientific evidence that the therapeutic effects of Nefang are as a result of the synergy between antiplasmodial and other pharmacological activities of its constituent plants, thereby justify the folk use of Nefang for the treatment malaria fever. This could be useful in its subsequent development for clinical application.
